# Comparative Effects of Pulsed Field and Radiofrequency Ablation on Blood Cell Parameters During Pulmonary Vein Isolation

**DOI:** 10.3390/biomedicines13081828

**Published:** 2025-07-25

**Authors:** Lucio Addeo, Federica Di Feo, Mario Vaccariello, Alfonso Varriale, Benedetta Brescia, Davide Bonadies, Stefano Nardi, Luigi Argenziano, Vittoria Marino, Vincenza Abbate, Luigi Cocchiara, Pasquale Guarini, Laura Adelaide Dalla Vecchia, Francesco Donatelli

**Affiliations:** 1 Department of Advanced Biomedical Sciences, University of Naples Federico II, 80131 Naples, Italy; 2 Pineta Grande Hospital, 81030 Castel Volturno, Italy; 3 Department of Medicine, Surgery and Dentistry, University of Salerno, 84084 Salerno, Italy; 4 U.O Cardiologia, Clinica Sanatrix, 80127 Naples, Italy; 5 Department of Cardiology, IRCCS Istituti Clinici Scientifici Maugeri, 20138 Milan, Italy; 6 Department of Clinical and Community Sciences, University of Milan, 20122 Milan, Italy

**Keywords:** atrial fibrillation, pulsed field ablation, pulmonary vein isolation, radiofrequency ablation, blood cell parameters, hemolysis, lactate dehydrogenase, post-procedural course

## Abstract

**Background:** Pulsed field ablation (PFA) is a novel non-thermal modality for pulmonary vein isolation (PVI) in atrial fibrillation (AF), offering myocardial selectivity through irreversible electroporation while sparing surrounding structures. However, concerns have emerged regarding potential subclinical hemolysis, reflected by alterations in biochemical markers such as lactate dehydrogenase (LDH). **Methods:** We conducted a retrospective, single-center study involving 249 patients undergoing PVI: 121 treated with PFA (PulseSelect or FARAPULSE) and 128 with radiofrequency (RF) ablation (PVAC catheter). Laboratory parameters were assessed at baseline, post-procedure, and at discharge, including hemoglobin, hematocrit, red blood cell (RBC) count, platelet count, creatinine, and LDH. The primary endpoint was the variation in blood cell indices; the secondary endpoint was the evaluation of LDH and hematocrit changes. Statistical analysis included *t*-tests and chi-square tests. **Results:** Baseline characteristics and pre-procedural labs did not differ significantly between groups. No significant changes in hemoglobin, hematocrit, RBC count, platelet count, or creatinine were observed post-ablation or at discharge. However, LDH levels significantly increased in the PFA group both post-procedurally and at discharge (*p* < 0.001), without concurrent changes in other blood cell parameters. **Conclusions:** PFA and RF ablation yield comparable hematological profiles after PVI, with no significant impact on key blood cell parameters. Nonetheless, the consistent rise in LDH levels in the PFA group suggests mild, subclinical hemolysis or tissue injury due to more extensive lesions. While supporting the hematologic safety of PFA, these findings underscore the need for further studies to assess the clinical significance of these biochemical alterations, particularly in high-risk patients or extensive ablation settings.

## 1. Introduction

Pulsed field ablation (PFA) has emerged as a groundbreaking technique for the treatment of atrial fibrillation (AF). Unlike conventional thermal ablation methods, such as radiofrequency or cryoablation, PFA delivers high-voltage, short-duration electrical pulses that induce irreversible electroporation in myocardial cells. This process selectively disrupts cardiomyocytes while preserving surrounding non-cardiac structures like the esophagus, phrenic nerve, and pulmonary veins, due to differential membrane susceptibility across tissue types. As a result, PFA has been positioned as a potentially safer and more anatomically precise alternative to thermal techniques, especially in challenging areas.

Despite its advantages, growing clinical evidence suggests that PFA may be associated with hemolysis, raising questions about its systemic safety profile. In a recent multicenter study, hemolysis was documented in over 94% of patients undergoing PFA, with reductions in serum haptoglobin and elevations in free hemoglobin, lactate dehydrogenase (LDH), and bilirubin levels. In contrast, only 6.8% of patients treated with conventional radiofrequency (RF) ablation exhibited comparable findings [1]. The severity of hemolysis was found to correlate with the number of PFA applications, indicating a dose-dependent relationship [1].

Another study comparing PFA and RF ablation reported that PFA led to significantly higher levels of red blood cell microparticles, LDH, and indirect bilirubin, along with a greater decline in haptoglobin. Interestingly, the magnitude of these changes was more pronounced in patients who received additional ablation beyond pulmonary vein isolation (PVI), underscoring the impact of ablation volume on hemolysis [2].

Although these biochemical alterations suggest a non-target effect of electroporation, clinical consequences appear limited. In the MANIFEST-17K registry, which included more than 17,000 PFA-treated patients, hemolysis-associated acute kidney injury requiring dialysis occurred in just 0.03% of cases, predominantly in patients with extensive ablation [3]. Current evidence points to hemolysis as a reproducible but largely subclinical phenomenon following PFA. Therefore, we conducted a comparative study between this novel energy modality and conventional thermal ablation to evaluate their respective impacts on blood cell parameters.

## 2. Materials and Methods

### 2.1. Study Population and Design of the Study

We conducted a single-center, retrospective study involving a total of 249 patients who underwent PVI between January 2023 and December 2024. Among them, 128 patients underwent PVI using thermal energy—specifically, RF ablation with the PVAC system (Medtronic, Minneapolis, MN, USA)—while the remaining 121 patients underwent PVI using PFA, utilizing either the PulseSelect system (Medtronic, USA) or the FARAPULSE System (Boston Scientific, Marlborough, MA, USA). All of these systems employ single-shot technology, thereby improving the methodological consistency and reliability of the comparison between groups. Inclusion criteria required patients to be between 18 and 80 years of age, clinically stable, and diagnosed with either paroxysmal or persistent AF. Exclusion criteria included permanent AF, severe comorbid conditions (including severe anemia with Hb < 10 g/dL or hematological disorders), recent stroke or transient ischemic attack, recent coronary or peripheral revascularization procedures, pregnancy, and any contraindication to anticoagulation therapy. Data were systematically collected on demographic and clinical variables, including age, gender, body mass index (BMI), left ventricular ejection fraction (LVEF), cardiovascular risk factors, and current pharmacological treatments, with particular attention to the use of anticoagulants and antiarrhythmic agents. Additionally, the volume of fluids infused during the procedure was recorded as well as the procedural time. The study was conducted in accordance with the ethical standards outlined in the Declaration of Helsinki.

### 2.2. Study Endpoints

The primary endpoint of the study was to assess the change in biochemical markers, including platelet count, hemoglobin, creatinine, and red blood cells (RBC) levels, measured before and after the procedure and compared between the two treatment groups. The secondary endpoint was the evaluation of either an increase in LDH or a decrease in hematocrit, also compared between the two groups.

### 2.3. Procedural Description and Periprocedural Management

All procedures were carried out by a single high-volume operator with >2000 prior PVI. Vascular access was obtained through the right and left femoral veins and the left femoral artery. A 7-Fr introducer was inserted into the left femoral vein to allow for backup pacing, while a 6-Fr introducer was placed in the femoral artery to facilitate hemodynamic monitoring. Transseptal puncture was performed using a long sheath, followed by the administration of unfractionated heparin to maintain an activated clotting time (ACT) of ≥250 ms. Ablation was then carried out using either the PFA or RF system, according to each system’s standardized protocol and regulatory approval, and only encircling of the veins was performed, without additional lesions. Specifically, regarding the P-VAC GOLD catheter, isolation for each pulmonary vein was achieved with 16 consecutive bipolar applications (20 W, maximum 60 °C), delivered circumferentially (four primary rotations with four energy deliveries per rotation) to provide 360° coverage of the venous ostium. Whenever real-time mapping revealed residual conduction gaps, targeted touch-up lesions were applied solely to the affected segment until endoluminal potentials were completely abolished. Each application lasted 60 s.

For the FARAPULSE system, each pulmonary vein was isolated with the pentaspline catheter sequentially deployed in basket and flower configurations. Eight pulse-train applications were typically delivered per vein, four in the basket configuration (two applications followed by two applications after a slight rotation) followed by four additional deliveries in the flower configuration (same protocol as basket configuration), to achieve complete circumferential coverage. Energy was delivered as bipolar, biphasic waveforms composed of ultra-short, microsecond-scale pulses. If residual conduction was observed on mapping, additional pulse trains were delivered at the site of persistent potentials until bidirectional block was confirmed.

Finally, for the PulseSelect system, PVI was obtained by positioning the multielectrode catheter at the ostium of each vein and delivering a series of pulsed-electric-field applications. Typically, four applications were delivered per vein, with minor rotations between deliveries to ensure circumferential coverage. Each application consisted of a train of high-voltage, biphasic pulses. In the presence of persistent electrical activity, additional applications were selectively administered at the site of residual conduction until isolation was confirmed.

All pulmonary veins were individually isolated, with successful isolation confirmed following the final energy application. At the end of the procedure, ACT was allowed to decrease to below 140 ms before sheath removal, and hemostasis was achieved using a compressive bandage. Anamnesis and list of medications were obtained at the time of admission, followed by baseline laboratory testing. Laboratory tests were then repeated after the procedure and again on the day of discharge. The amount of fluid infused and the procedural time were recorded immediately after the procedure and documented in the corresponding procedural report.

### 2.4. Statistical Analysis

Data were statistically analyzed using SPSS version 25 (SPSS Inc., Chicago, IL, USA). Continuous variables were expressed as mean ± standard deviation, while categorical variables were presented as counts and percentages. Comparisons between continuous variables were performed using the independent samples *t*-test. Categorical variables were compared using the chi-square test or Fisher’s exact test, as appropriate. Patient characteristics and laboratory test results were thoroughly documented and analyzed. All collected data were compared between the two treatment groups to allow for a comprehensive evaluation of potential risk factors and to identify or exclude possible confounding variables.

## 3. Results

### 3.1. Baseline Characteristics

Baseline characteristics are summarized in Table 1. No significant differences were observed between the two groups with respect to the demographic or clinical variables considered. Similarly, pre-procedural laboratory values did not differ significantly between groups, as shown in Table 2. The only significant difference recorded was in procedural time.

### 3.2. Study Endpoints

#### 3.2.1. Primary Endpoints

**Figure 1:**
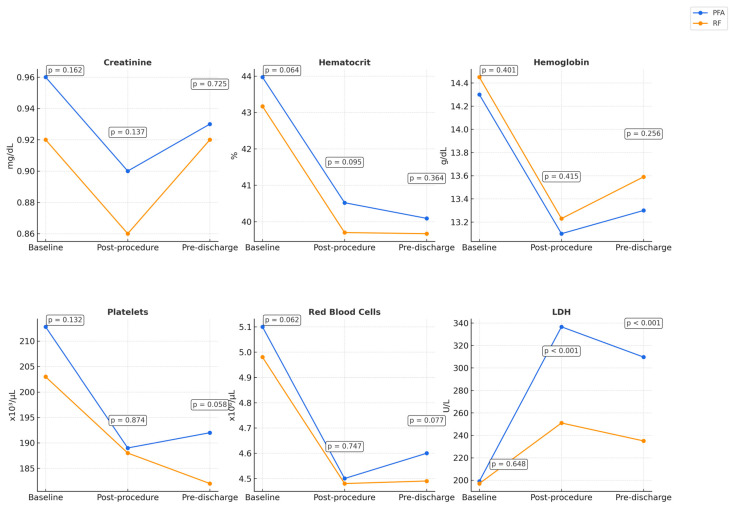
Changes in blood cell and biochemical parameters post-ablation in PFA and RF groups. The reported *p*-values reflect intergroup comparisons at the specified time points.

Regarding the primary endpoint, no statistically significant differences were observed in blood cell parameters (including hemoglobin, RBC count, platelet count, and creatinine levels) when measured both post-procedure and at the time of hospital discharge. Figure 1 shows the primary endpoints.

#### 3.2.2. Secondary Endpoints

Regarding secondary endpoints, again no statistically significant differences were observed in hematocrit levels after the procedure and at the time of hospital discharge. Conversely, a statistically significant difference was observed in LDH levels between the two groups (*p* < 0.001). (Table 3). Figure 1 shows the secondary endpoints and illustrates the changes observed in key hematologic and biochemical parameters.

## 4. Discussion

Procedural technique, energy settings, and lesion characteristics were harmonized across platforms to allow for a like-for-like energy-level comparison. No linear or focal lines beyond the pulmonary vein ostia were created, thereby limiting lesion heterogeneity. Energy parameters were fixed at the manufacturer-recommended “standard-dose” settings: 16 × 20 W phased-RF applications per vein for PVAC-GOLD, 8 high-voltage (2.0 kV) pulse trains per vein for FARAPULSE, and 4 pulse trains (1.5 kV) per vein for PulseSelect. These distinct biophysical mechanisms generate different lesion morphologies (coagulative and ~4 mm deep with phased-RF versus sharply demarcated, non-thermal electroporation zones with PFA). Using a standardized technique and energy delivery, we minimized inter-procedural variability, and this should ensure that any biochemical divergence reflects the intrinsic effects of the energy modality itself. In this retrospective analysis of patients undergoing PVI with PFA or RF ablation, we did not observe a statistically significant reduction in creatinine, hemoglobin, hematocrit, platelet count, or RBC count following the procedure or at hospital discharge. These findings are noteworthy, particularly given the emerging body of literature suggesting a mild but reproducible degree of hemolysis following PFA, especially when a high number of applications have been delivered [4], although generally not associated with clinical signs of hemolytic anemia or hemoglobinuria [1,2]. While a modest reduction in all major blood cell parameters was observed post-procedurally, the pattern was consistent across both treatment groups. This trend may reflect non-specific procedural factors such as intravascular volume expansion and fluid administration rather than an energy-specific cytotoxic effect. Although the PFA procedures were shorter than the RF procedures, consistent with current evidence [5], the higher infusion rate used during PFA meant that the total fluid volume ultimately administered was essentially comparable in both groups. Popa et al. have suggested that these mild declines are more likely attributable to procedural hemodilution rather than hemolysis per se [1]. Regarding hemoglobin levels, Popa et al. reported that both PFA and RFA were associated with a slight decrease in hemoglobin, hematocrit, and RBCs with no statistically significant differences between mean values of these markers. While a higher relative hemoglobin decrease (Δ −0.83 ± 1.1 versus −0.50 ± 0.9; *p* = 0.036) and a higher incidence of hemoglobin drop ≥2 mg/dL were found with PFA (21/113 [18.6%; 95% CI, 11.9–27.0%] versus 3/66 [4.6%; 95% CI, 0.95–12.7%], a ≥2 g/dL decline in hemoglobin was observed in 18.6% of patients treated with PFA compared to only 4.6% in the RFA group [1]. Our findings did not show significant differences in the two treatment groups, in line with other studies reporting mild hemolysis without a significant drop in hemoglobin following PVI with PFA [6]. This observation is also confirmed in comparative studies with RF ablation [2]. In our cohort, hemoglobin levels remained similar between groups at both post-procedural and discharge time points, without reaching statistical significance. Similarly, platelet counts in our population showed a mild non-significant decrease post-ablation, consistent across both groups. This suggests an absence of substantial platelet activation or consumption. These findings are in agreement with those of Osmancik et al., who observed that, despite a significantly higher release of troponin I following PFA compared to RF ablation, there were no significant differences in platelet activation or coagulation cascade activation between the two modalities. Additionally, the inflammatory response was slightly lower in the PFA group [7]. Serum creatinine also showed unchanged levels both after the procedure and at the time of discharge. This is consistent with current evidence, as no significant changes have been observed, regardless of the type of energy applied (PFA or RF) [8]. However, current evidence highlights a post-ablation creatinine increase >5 µmol/L occurring more frequently in the PFA group (33/124 [26.6%; 95% CI, 19.1–35.3%] versus 8/60 [13.3%; 95% CI, 5.9–24.6%]; *p* = 0.042), with a potential (but not significant) increase in acute kidney injury incidence [2]. Hematocrit and RBC counts also demonstrated slight post-procedural declines in both groups. Again, this pattern is likely due to procedural hemodilution rather than marked hemolysis. Nonetheless, it is important to acknowledge that while standard hematological indices remained stable, PFA has been associated with a more pronounced biochemical profile of hemolysis in several studies [9,10]. Specifically, elevations in red blood cell microparticles, LDH, and indirect bilirubin, along with a decrease in haptoglobin, have been documented following PFA, indicating a certain degree of subclinical intravascular hemolysis relative to thermal ablation [3]. In our study, although we documented an isolated post-procedural rise in LDH, there was no accompanying fall in hemoglobin or RBC count. This pattern points toward LDH release from muscle or other tissue trauma during the ablation rather than overt hemolysis, yet a mild or subclinical hemolytic component cannot be definitively excluded with the data at hand. Future studies that include LDH isoenzyme profiling and haptoglobin measurements will be necessary to discriminate between tissue leakage and RBCs destruction as the principal source of the LDH increase. Recent evidence has suggested that LDH elevation following PFA correlates with the number of applications delivered during PVI, supporting the idea of a dose-dependent effect [11]. Similar associations have also been reported between the number of PFA pulses and the occurrence of hemoglobinuria or acute kidney injury [12]. Importantly, whether these subclinical changes in blood cell parameters have clinical relevance remains unclear. While our findings support the hematological safety of PFA when used in standard and strict PVI protocols, including only PVI without additional lesions, further investigation is warranted, particularly in vulnerable populations or in procedures involving extensive ablation. The primary limitations of our study include its retrospective design, single-center nature, and lack of long-term hematological follow-up. Future prospective, multicenter studies are needed to clarify the potential dose–response relationship between the number of PFA applications and the extent of hematological alterations, and whether these changes hold prognostic or procedural significance.

## Figures and Tables

**Table 1:** Baseline characteristics in the two groups.

	PFA (n = 121)	RF (n = 128)	*p*-Value
Males (%)	60.33% [51.4–68.6]	64.06% [55.5-71.9]	0.634
Age	71 ± 11.9 [69.37–73.61]	70 ± 10.2 [67.97–71.50]	0.478
BMI	26.86 ± 4.08 [26.13–27.59]	27.01 ± 3.65 [26.38–27.25]	0.761
Current Smoker (%)	18.18% [12.3–26.0]	20.31% [14.3–28.1]	0.791
Arterial Hypertension (%)	52.89% [44.0–61.6]	53.91% [45.3–62.3]	0.974
Diabetes (%)	10.74% [6.4–17.5]	10.16% [6.0–16.6]	1.000
LVEF (%)	57 ± 2.82 [56.66–57.66]	57.66 ± 3.32 [57.09–58.24]	0.092
ACE/ARBs/ARNI (%)	48.76% [40.0–57.6]	53.13% [44.5–61.6]	0.574
Diuretics (%)	2.48% [0.8–7.0]	7.03% [3.7–12.8]	0.138
β-Blockers (%)	51.24% [42.4–60.0]	57.81% [49.2–66.0]	0.361
Statins (%)	13.22% [8.3–20.4]	17.97% [12.3–25.5]	0.392
Antiarrhythmic Drug Class I/III/IV (%)	64.46% [55.6–72.4]	68.75% [60.3–76.1]	0.560
Oral Anticoagulation (%)	89.26% [82.5–93.6]	89.84% [83.4–94.0]	1.000
Antiplatelet (%)	0	1.56% [0.4–5.5]	0.498
Liquid infusion (ml)	731 ± 111 [711.59–751.14]	757 ± 112 [737.74–776.46]	0.067
Procedural Time	58.34 ± 7.64 [56.96–59.72]	77.23 ± 11.47 [75.22–79.24]	<0.001

BMI: Body Mass Index; LVEF: Left Ventricular Ejection Fraction; ACE: Angiotensin-Converting Enzyme; ARBs: Angiotensin Receptor Blockers; ARNI: Angiotensin Receptor Neprilysin Inhibitors.

**Table 2:** Laboratory testing variables at the time of hospital admission.

	PFA (n = 121)	RF (n = 128)	*p*-Value
Creatinine (mg/dL)	0.96 ± 0.26 [0.91–1.0]	0.92 ± 0.18 [0.89–0.95]	0.162
Hematocrit (%)	43.97 ± 3.51 [43.37–44.61]	43.17 ± 3.27 [42.61–43.74]	0.064
Hemoglobin (g/dL)	14.3 ± 1.46 [14.03–14.55]	14.45 ± 1.35 [14.21–14.68]	0.401
Platelets (×10^9^/L)	212.8 ± 42.27 [205.05–220.21]	203 ± 59 [192.31–212.96]	0.132
RBC (×10^12^/L)	5.1 ± 0.51 [4.97–5.16]	4.98 ± 0.50 [4.9–5.07]	0.062
LDH (U/L)	199 ± 35 [193.06–205.72]	197 ± 34 [190.55–202.52]	0.648

RBC: Red Blood Cells; LDH: Lactate Dehydrogenase.

**Table 3:** Post-procedural and pre-discharge laboratory testing variables.

	PFA (n = 121)	RF (n = 128)	*p*-Value
Creatinine post-procedure (mg/dL)	0.90 ± 0.24 [0.86–0.94]	0.86 ± 0.18 [0.83–0.90]	0.137
Creatinine pre-discharge (mg/dL)	0.93 ± 0.24 [0.89–0.97]	0.92 ± 0.21 [0.88–0.96]	0.725
Hematocrit post- procedure (%)	40.52 ± 4.17 [39.80–41.28]	39.70 ± 3.57 [39.08–40.32]	0.095
Hematocrit pre-discharge (%)	40.09 ± 3.30 [39.53–40.71]	39.67 ± 4.06 [38.96–40.38]	0.364
Hemoglobin post- procedure (g/dL)	13.1 ± 1.17 [12.89–13.30]	13.23 ± 1.37 [13.00–13.47]	0.415
Hemoglobin pre-discharge (g/dL)	13.3 ± 1.18 [13.06–13.48]	13.59 ± 2.67 [13.12–14.05]	0.256
Platelets post- procedure (×10^9^/L)	189 ± 42 [181.78–196.83]	188 ± 58 [177.79–197.83]	0.874
Platelets pre-discharge (×10^9^/L)	192 ± 33 [186.26–198.17]	182 ± 50 [173.75–191.13]	0.058
RBC post- procedure (×10^12^/L)	4.5 ± 0.48 [4.44–4.61]	4.48 ± 0.51 [4.39–4.57]	0.747
RBC pre-discharge (×10^12^/L)	4.6 ± 0.45 [4.56–4.72]	4.49 ± 0.54 [4.40–4.59]	0.077
LDH post- procedure (U/L)	336.6 ± 129.47 [312.72–358.86]	251 ± 50 [241.80–259.28]	**<0.001**
LDH pre-discharge (U/L)	309.6 ± 113.74 [289.89–330.39]	235 ± 53 [226.14–244.51]	**<0.001**

## Data Availability

The original contributions presented in this study are included in the article. Further inquiries can be directed to the corresponding author (addeolucio@gmail.com).

## Abbreviations

The following abbreviations are used in this manuscript:

ACT	Activated Clotting Time
AF	Atrial Fibrillation
BMI	Body Mass Index
LDH	Lactate Dehydrogenase
LVEF	Left Ventricular Ejection Fraction
PFA	Pulsed Field Ablation
PVI	Pulmonary Vein Isolation
RBC	Red Blood Cell
RF	Radiofrequency
